# Obligate Funic Presentation: A Case Report of Velamentous Cord Insertion in a Normally Implanted Placenta

**DOI:** 10.1055/a-2848-3148

**Published:** 2026-06-24

**Authors:** Amberly Lao, Gabrielle Shuman, Julia Kim, Erin Conroy, Martin Chavez, Patricia Rekawek

**Affiliations:** 1 Department of Obstetrics and Gynecology 24998 NYU Langone Hospital—Long Island, NYU Long Island School of MedicineMineolaNew YorkUnited States; 2 Department of Obstetrics and Gynecology 8784 University of California San DiegoLa JollaCaliforniaUnited States

**Keywords:** velamentous, cord prolapse, funic presentation, cord insertion

## Abstract

Funic presentation refers to umbilical cord loops positioned between the presenting fetal part and the internal cervical os. While often transient, persistent positioning due to anatomic predisposition represents a high-risk variant and a potential precursor to cord prolapse. We report the case of a 39-year-old pregnant woman with a velamentous cord insertion in whom the umbilical cord remained persistently positioned over the cervical os, a presentation we term “obligate funic presentation.” The patient’s prenatal care was uncomplicated, and an ultrasound at 36 weeks identified umbilical cord loops over the internal os. She was admitted for close monitoring and subsequently developed regular preterm contractions with cervical change and recurrent variable decelerations that persisted despite resuscitative efforts. A cesarean delivery was performed at 36 weeks without evidence of cord prolapse at the time of delivery. A healthy male infant was delivered, who did not require neonatal intensive care unit (NICU) admission. Placental pathology revealed a marginal cord insertion. This case highlights an obligate funic presentation occurring in the absence of placenta previa when the cord insertion is velamentous or marginal. Early recognition is critical, as this uncommon but high-risk condition may predispose to cord compression or prolapse, necessitating heightened surveillance and individualized delivery planning and patient counseling.

## Case Report

**Video 1** Transvaginal sagittal view with color Doppler at 36 weeks’ gestation, revealing multiple loops of umbilical cord overlying the internal cervical os.


Funic presentation, also known as cord presentation, refers to the presence of free umbilical cord loops between the presenting fetal part and the internal cervical os. This is an uncommon finding, occurring in 0.006 to 0.16% of term pregnancies. 1 It may serve as a precursor to cord prolapse—an obstetric emergency requiring cesarean delivery to reduce perinatal morbidity and mortality. 2 3 4 While funic presentation is generally considered a transient finding on prenatal ultrasonography, 4 an obligate funic presentation occurs when the umbilical cord persistently remains between the presenting fetal part and cervix, due to anatomic entrapment. 5 6

This anatomic entrapment may arise from structural or placental factors, such as a low-lying placenta or placenta previa, which restrict upward retraction of the cord, or a velamentous or marginal cord insertion, which positions the cord close to the cervix. 5 6 7 A velamentous cord insertion specifically refers to umbilical vessels traversing the membranes without the protection of Wharton’s jelly, predisposing not only to obligate funic presentation, but also to cord compression and rupture. 8 There is no standardized approach to the management of prenatally detected funic presentation, as existing data are limited to case reports. We present a case of an obligate funic presentation associated with a velamentous cord insertion and a normally implanted placenta. Persistent loops of umbilical cord remained anchored over the internal os, creating a constant funic presentation and a significant risk of acute cord prolapse at membrane rupture.

**Fig. 1:**
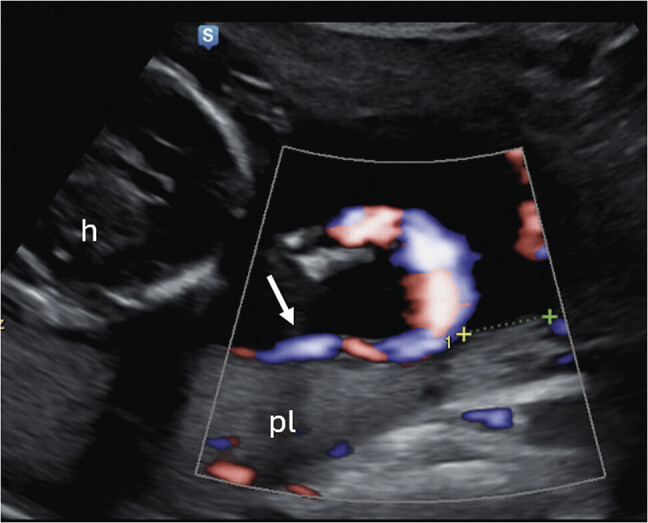
Transabdominal scan in sagittal plane with color Doppler at 20 weeks’ gestation demonstrating velamentous cord insertion of a posterior placenta. Umbilical vessels traverse the membranes before reaching the placental edge. h, head; pl, placenta; arrow points to umbilical cord.

**Fig. 2:**
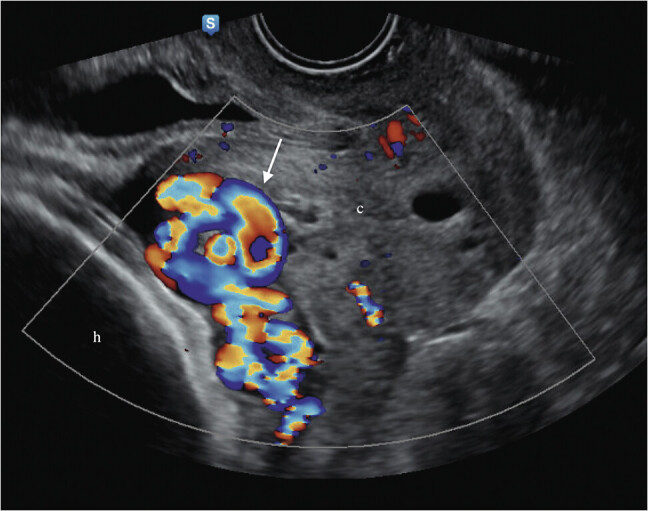
Transvaginal scan in sagittal plane with color Doppler at 36 weeks’ gestation showing loops of umbilical cord overlying the internal cervical os. h, head; cx, cervix; arrow points to umbilical cord.

A 39-year-old gravida 3 para 2 with one prior vaginal delivery and one prior cesarean section underwent an anatomy ultrasound at 20 weeks, which demonstrated a posterior placenta with velamentous cord insertion ( Fig. 1 ). She underwent serial sonographic surveillance every 4 weeks. Her pregnancy had been uncomplicated, and at the 36-week ultrasound, multiple free loops of umbilical cord were visualized directly overlying the internal cervical os ( Fig. 2 ). The cervix measured 3.4 cm in length, without funneling or dynamic changes. Fetal growth and amniotic fluid volume were appropriate for gestational age. Several repeated real-time assessments during the same visit confirmed that the cord persisted in this position without movement ( Video 1 ). Given the persistent funic presentation, the patient was referred to labor and delivery for continuous fetal monitoring and repeat imaging. Follow-up sonography again showed an unchanged funic presentation with no interval retraction of the cord.

During monitoring, the fetus developed recurrent variable decelerations consistent with intermittent cord compression, which persisted despite resuscitative measures. Because of the persistent cord position and evolving fetal heart rate abnormalities, a decision was made to proceed with cesarean delivery at 36 ^1/7^ weeks. Intraoperative findings included an intact amniotic sac with umbilical cord noted immediately after delivery of the infant’s head, without evidence of cord prolapse. The placenta demonstrated a marginal cord insertion on pathology. The neonate was delivered with Apgar scores of 9 and 9 at 1 and 5 minutes, respectively, and did not require neonatal intensive care unit (NICU) admission. The postpartum course was unremarkable, with no maternal or neonatal complications.

## Discussion

Recognition of funic presentation during prenatal ultrasound permits individualized patient counseling, delivery planning, and a discussion of the possible risks associated with it, including cord prolapse. Although large series are lacking, one cohort reported cord prolapse in approximately 1 in every 13 pregnancies with antenatally diagnosed funic presentation. 4

In this case, the persistence of the umbilical cord at the cervical os reflected an anatomic tethering effect from the low-lying velamentous insertion, which limited the cord’s ability to retract cranially. We propose the term “obligate” funic presentation to describe cases in which the umbilical cord remains persistently positioned between the fetal presenting part and cervix due to structural or placental factors. Because spontaneous resolution is unlikely, these cases should be managed as high-risk, with individualized surveillance and delivery planning. Additionally, we advocate for multidisciplinary delivery planning that includes maternal–fetal medicine specialists and the delivering OBGYN.

When predisposing conditions such as low-lying placenta, placenta previa, or velamentous or marginal cord insertion are detected, serial sonography can facilitate early identification of cord presentation. Previous reports describe funic presentation identified at 27 weeks in the setting of low-lying placentas with marginal insertions, with cesarean deliveries at 31 and 35 weeks for recurrent variable decelerations. 7 9 Likewise, velamentous insertion alone has been associated with intermittent decelerations and increased likelihood of emergent cesarean delivery, particularly when located in the lower uterine segment. 10

In our patient, funic presentation developed later, at 36 weeks, despite a normally positioned placenta. The combination of persistent cord position, preterm contractions, and nonreassuring fetal heart rate tracings prompted cesarean delivery in the late-preterm period. This case illustrates that obligate funic presentation can occur even without placenta previa and supports early cesarean delivery once the diagnosis is established and signs of labor or fetal compromise appear, to reduce the risk of cord prolapse and adverse perinatal outcomes.

## References

[1] Ezra Y Strasberg S R Farine D Does cord presentation on ultrasound predict cord prolapse? Gynecol Obstet Investig 2003 56 01 6 9 12867760 10.1159/000072323

[2] Jones G Grenier S Gruslin A Sonographic diagnosis of funic presentation: implications for delivery BJOG 2000 107 08 1055 1057 10955444 10.1111/j.1471-0528.2000.tb10415.x

[3] Lange I R Manning F A Morrison I Chamberlain P F Harman C R Cord prolapse: is antenatal diagnosis possible? Am J Obstet Gynecol 1985 151 08 1083 1085 3885744 10.1016/0002-9378(85)90388-6

[4] Vintzileos A M Nochimson D J Lillo N L Tohan N Ultrasonic diagnosis of funic presentation J Clin Ultrasound 1983 11 09 510 511 6417191 10.1002/jcu.1870110913

[5] Sapantzoglou I Psarris A Diamantopoulou P Persistent funic presentation and sonographic assessment of the risk for umbilical cord prolapse Ultrasound Int Open 2023 9 01 E33 E35 38099217 10.1055/a-2097-5143 PMC10721356

[6] Kusakabe H Miyamoto T Yasuda E Persistent cord presentation due to marginal cord insertion at the lower edge of a low-lying placenta: a case of successful vaginal delivery Cureus 2025 17 05 e84467 40539168 10.7759/cureus.84467 PMC12178148

[7] Oyelese Y Yeo L Kinzler W Smulian J Vintzileos A M Persistent funic presentation resulting from marginal cord insertion into a low-lying placenta Ultrasound Obstet Gynecol 2004 24 06 692 693 15386613 10.1002/uog.1069

[8] Mimura K Endo M Matsuzaki S Tomimatsu T Kimura T Persistent funic presentation due to velamentous cord insertion adjacent to the internal os but not vasa previa Taiwan J Obstet Gynecol 2020 59 01 167 168 32039791 10.1016/j.tjog.2019.10.001

[9] Uchide K Ueno H Inuyama R Murakami K Terada S Cord presentation with posterior placenta praevia Lancet 1997 350 9089 1448 10.1016/S0140-6736(05)64209-2 9371174

[10] Hasegawa J Matsuoka R Ichizuka K Sekizawa A Farina A Okai T Velamentous cord insertion into the lower third of the uterus is associated with intrapartum fetal heart rate abnormalities Ultrasound Obstet Gynecol 2006 27 04 425 429 16479618 10.1002/uog.2645

